# Measles in the 21st Century: Progress Toward Achieving and Sustaining Elimination

**DOI:** 10.1093/infdis/jiaa793

**Published:** 2021-09-30

**Authors:** Paul A Gastañaduy, James L Goodson, Lakshmi Panagiotakopoulos, Paul A Rota, Walt A Orenstein, Manisha Patel

**Affiliations:** 1 Division of Viral Diseases, National Center for Immunization and Respiratory Diseases, Centers for Disease Control and Prevention, Atlanta, Georgia, USA; 2 Global Immunization Division, Center for Global Health, Centers for Disease Control and Prevention, Atlanta, Georgia, USA; 3 Immunization Safety Office, National Center for Emerging and Zoonotic Infectious Diseases, Centers for Disease Control and Prevention, Atlanta, Georgia, USA; 4 Emory University and the Emory Vaccine Center, Atlanta, Georgia, USA

**Keywords:** measles, measles, mumps, rubella vaccine, MMR, elimination, eradication

## Abstract

The global measles vaccination program has been extraordinarily successful in reducing measles-related disease and deaths worldwide. Eradication of measles is feasible because of several key attributes, including humans as the only reservoir for the virus, broad access to diagnostic tools that can rapidly detect measles-infectious persons, and availability of highly safe and effective measles-containing vaccines (MCVs). All 6 World Health Organization (WHO) regions have established measles elimination goals. Globally, during 2000–2018, measles incidence decreased by 66% (from 145 to 49 cases per million population) and deaths decreased by 73% (from 535 600 to 142 300), drastically reducing global disease burden. Routine immunization with MCV has been the cornerstone for the control and prevention of measles. Two doses of MCV are 97% effective in preventing measles, qualifying MCV as one of the most effective vaccines ever developed. Mild adverse events occur in <20% of recipients and serious adverse events are extremely rare. The economic benefits of measles vaccination are highlighted by an overall return on investment of 58 times the cost of the vaccine, supply chains, and vaccination. Because measles is one of the most contagious human diseases, maintenance of high (≥95%) 2-dose MCV coverage is crucial for controlling the spread of measles and successfully reaching measles elimination; however, the plateauing of global MCV coverage for nearly a decade and the global measles resurgence during 2018–2019 demonstrate that much work remains. Global commitments to increase community access to and demand for immunizations, strengthen national and regional partnerships for building public health infrastructure, and implement innovations that can overcome access barriers and enhance vaccine confidence, are essential to achieve a world free of measles.

Measles is a febrile rash illness that can lead to serious complications and death and one of the world’s most contagious viral diseases. The basic reproduction number (or average number of secondary cases generated by an infectious person in a fully susceptible population) for measles is estimated to be 12–18, higher than that of many other common childhood illness (eg, influenza, pertussis). Under the assumption of a homogenously mixing population, such high transmissibility means that significantly high population immunity levels of >92%–94% are needed to impede sustained measles virus transmission. Measles vaccines have been enormously successful in controlling measles globally, demonstrating the feasibility of reaching a measles eradication goal (ie, reduction of measles cases globally to zero). In the current article, we review the significant impact measles vaccination uptake has had on reducing measles disease burden worldwide, the origin of measles vaccines and their safety and effectiveness profiles, global trends in vaccination coverage, the economic benefits of investing in measles vaccination, the various setbacks encountered in measles control in recent years, and key challenges that must be overcome to achieve a world free of measles.

## MEASLES DISEASE BURDEN AND IMPACT OF MEASLES VACCINATION

Before the introduction of measles vaccination, measles caused substantial human disease and death worldwide, infecting nearly everyone by 15 years of age. Measles was common in all parts of the world and caused an estimated 135 million cases and more than 6 million deaths globally each year [1]. In the United States alone, an estimated 3–4 million people acquired measles every year (roughly equivalent to a birth cohort), of which approximately 500 000 cases and nearly 500 deaths were reported annually [2].

Although most persons fully recover from measles without sequelae, the disease entails significant morbidity and mortality risks. Common complications of measles include otitis media and diarrhea, but more serious complications can also occur and include pneumonia, encephalitis, and subacute sclerosing panencephalitis, a slowly progressive neurologic sequela of measles that is universally fatal (estimated risk, 1 subacute sclerosing panencephalitis case per 5000 measles cases) [3]. Secondary bacterial infections related to measles-induced immunosuppression (inhibition of lymphocyte proliferation and decrease in specific preexisting antibodies) can further complicate disease progression and recovery [4–8]. Measles case fatality ratios (CFRs) vary widely, depending on access to quality healthcare and the underlying nutritional and health status of those infected [9]. Measles CFRs in high-income countries such as the United States can be as low as 0.1% (or lower), but are much higher in other settings; CFRs have been estimated to be 4%–5% in Africa, and as high as 30% among vulnerable children during humanitarian crises [10].

**Figure 1.**
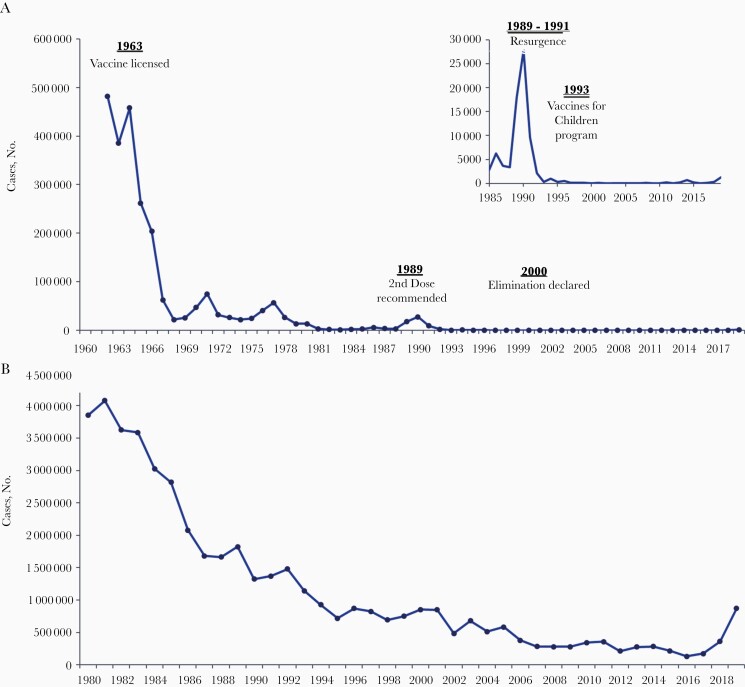
Number of reported measles cases in the United States from 1962 to 2019 (*A*) and worldwide from 1980 to 2019 (*B*). Data from US Centers for Disease Control and Prevention *Morbidity and Mortality Weekly Report*; global data available at http://apps.who.int/immunization_monitoring/globalsummary/timeseries/tsincidencemeasles.html.

After more widespread use of measles vaccines globally in the 1980s, measles incidence and mortality rates decreased to low levels in all regions of the world [11]. During 2000–2018, the worldwide annual reported measles incidence per million population decreased by 66%, from 145 to 49 cases, the annual number of reported measles cases decreased by 59%, from 853 479 to 353 236 (Figure 1), and annual estimated measles deaths decreased by 73%, from 535 600 to 142 300 [11]. In the United States, where measles elimination (ie, absence of continuous endemic measles virus transmission for more than a year) was achieved in 2000, new measles cases originate through measles introductions from abroad, mainly from unvaccinated US travelers becoming infected and returning home with measles. During 2001–2018, a median of 79 total cases (range, 37–667), including a median of 28 internationally imported cases (range, 18–82 importations), were reported annually in the United States, with 3 confirmed measles-related deaths reported during that 18-year period [12, 13]. This low burden of measles equates to more than a 99% decline in the reported number of cases and deaths due to measles when comparing US prevaccine and postelimination periods (Figure 1) [14].

As of 2019, a total of 83 countries have verified measles elimination, paving a path forward for global eradication. Several key attributes position measles as a prime candidate for eradication: (1) absence of a nonhuman reservoir (ie, unimmunized humans are essential for the life cycle of the measles virus), (2) availability of practical and sensitive diagnostic tools, such as immunoglobulin (Ig) M serologic assays and molecular tests to identify persons with acute measles, and, most importantly, (3) highly effective measles vaccines. Measles eradication is feasible [15]; however, global commitment is essential to sustain longstanding progress to reduce measles morbidity and mortality rates and to achieve regional elimination goals.

## GENOTYPIC VARIATION AND PATHOGENESIS OF MEASLES VIRUS

Measles virus is a negative sense, single-stranded RNA virus with a genome size of 15 894 nucleotides [16]. Measles virus is considered monotypic (ie, a genus with only a single species); however, multiple genetically distinct lineages of wild-type measles virus have been described [17]; the WHO currently recognizes 24 genotypes [18].

Measles is characterized by a generalized maculopapular skin rash, fever above 38.3°C (101°F), and cough, coryza, and/or conjunctivitis. During the incubation period, the virus replicates in alveolar macrophages and dendritic cells before establishing a systemic infection in which infected lymphocytes disseminate the virus to major organ systems, peripheral tissues, including the skin, the respiratory tract and epithelial cells. In uncomplicated measles cases, clinical signs start to subside a few days after rash onset, and patients develop a robust immune response that mediates recovery and provides lifelong immunity [16].

## MEASLES VACCINE HISTORY AND DEVELOPMENT

Measles vaccines are attenuated (or weakened) measles viruses, so that infection through vaccination of immunocompetent persons leads to replication and immunity, but not disease. Data suggest that genotype A was widely distributed in the prevaccine era when the progenitors of the vaccine strains were first isolated; however, only a few measles viruses from the 1950s and 1960s have been available for sequencing. All live attenuated vaccine strains used in measles vaccines are members of genotype A.

Most of the measles vaccine strains currently in use were derived from the prototype Edmonston strain (Edmonston wild-type) which was isolated by Enders and Peebles in 1954 [19]. In addition to the live attenuated vaccine strains, a formalin-inactivated Edmonston vaccine was in use during 1963–1967; however, use of this vaccine was discontinued because vaccinated individuals were at risk for developing atypical measles after wild-type measles virus infection. Atypical measles is caused by antigen–antibody immune complex deposition and characterized by high fever, abdominal pain, myalgias, pneumonitis, and a petechial or vesicular and edematous rash. The syndrome is preventable by vaccination with a live-attenuated vaccine [20]. To develop live-attenuated vaccines, the Edmonston strain was passaged in human amnion and human kidney cells before being adapted to chicken embryo fibroblasts to generate the commonly used Moraten and Schwarz strains. Several vaccine strains were derived from other wild-type measles virus isolates, including the Leningrad-16 strain used in Russia, the Shanghai-191 strain used in China, and the CAM-70 strain used in multiple countries. All measles vaccines are produced in chicken embryo fibroblasts, except the Edmonston-Zagreb strain (from the Serum Institute of India), which was derived by further passage in human diploid cells, MRC-5 [20].

Comparison of the genomic sequences of nine measles vaccine strains with the sequence of the Edmonston wild-type virus has shown a relatively small amount of genetic heterogeneity. Although nucleotide substitutions were found in the noncoding and protein coding regions of the genomes, the overall genetic organization of the vaccine strains was conserved [21], and there are no clear genetic markers for attenuation. The biologic basis for attenuation may be the result of use of different cellular receptors by vaccine and wild-type viruses. Vaccines strains can use both CD46 and human signaling lymphocyte marker for entry, while wild-type viruses recognize only human signaling lymphocyte marker. Vaccine and wild type viruses use nectin 4 to infect epithelial cells before viral shedding [16, 22]. Importantly, the high attenuation of measles vaccine viruses impedes human-to-human transmission of these viruses; a systematic review of 773 articles including genotyping of virus strains in close contacts of vaccinated individuals found that all cases of measles in close contacts were due to wild-type virus [23].

The only wild-type measles viruses currently detected in circulation are members of genotypes D8, D4, B3, and H1. Although some antigenic variation between wild-type and vaccine strains has been described [24], the necessity for conservation of the receptor-binding domains on the viral hemagglutinin (H) protein, the surface glycoprotein that is the target of neutralizing antibodies, constrains antigenic drift (ie, accumulation of mutations in virus-surface proteins). This conservation of the H protein allows measles vaccines to be highly effective against all wild-type viruses, directly contributing to overall success of the global measles vaccination program, and likely cessation of transmission of 20 of the 24 known genotypes [25].

Vaccination elicits long-lived humoral and cellular immune responses. Serologic correlates for protection from measles virus have been established and a titer >120 mUI/mL is considered protective [26]. Most vaccination studies rely on measurement of the concentration of neutralizing antibodies in serum samples as a surrogate for the immune response [27].

## MEASLES VACCINE SAFETY

The safety of MCVs, including measles-only, measles-rubella (MR), measles-mumps-rubella (MMR), and MMR-varicella (MMRV) vaccines is well-established. MCVs have similar safety profiles and are well tolerated, and common reactions after vaccination are mild [20, 28]. Common adverse events after MMR vaccination depend on components of the vaccine and include fever (5%–15%), rashes (5%), and lymphadenopathy (5%–20%), as well as parotitis and transient arthralgias/arthritis [29–32]. These adverse events occur approximately 6–12 days after vaccination, the time period of peak vaccine virus replication. Compared with the first dose of vaccine, adverse events are less common after the second dose of vaccine, because most children are immune at the time they receive the second vaccination, and therefore, less viral replication occurs [33].

MMR vaccination is only rarely associated with serious adverse events, and both precautions and contraindications to MMR vaccination have been carefully delineated to minimize serious reactions. A review by the Institute of Medicine assessed whether there was both epidemiologic and mechanistic evidence of serious adverse events associated with MMR vaccine [34]. This review concluded that there is an increased risk of febrile seizures following MMR vaccine, and that the evidence supported a causal relationship between MMR vaccine and febrile seizures. The review also concluded a causal relationship between MMR vaccine and anaphylaxis, and MMR vaccine and MIBE in individuals with demonstrated immunodeficiencies.

The attributable risk of febrile seizures after MMR vaccine has been evaluated in multiple large population-based studies to be approximately 25–34 additional febrile seizures per 100 000 children vaccinated, and occurs most commonly 7–14 days after vaccination [35]. The risk is approximately twice as high for children aged 12–23 months who receive the MMRV combination vaccine compared with those who receive MMR and varicella vaccines separately [36]. These studies found that there was 1 additional febrile seizure per every 2300–2600 MMRV vaccine doses given, compared with MMR and varicella vaccines given separately. There was no increased risk in individuals receiving a second dose of MMRV compared to MMR and varicella separately at the recommended 4–6-year-old age range [37]. Based on these findings, providers are recommended to discuss the benefits and risks of vaccination with MMRV versus MMR and varicella separately for the first dose of measles vaccine, and to administer MMRV as the first dose only if the parent or caregiver expresses a preference for MMRV vaccine [28].

Thrombocytopenia can occur after natural measles infection, and a causal relationship between MMR vaccine and thrombocytopenia has been established based on observational studies, case reports, and biologic plausibility [34]. A large US study evaluating the risk of immune thrombocytopenic purpura (ITP) in children aged 12–23 months in the 6 weeks after MMR vaccination [38] found that 76% of ITP cases in children in this age-group were attributable to the MMR vaccine, and estimated a rate of 1 case of ITP per 40 000 vaccine doses given. Thrombocytopenia associated with vaccination was generally mild and resolved within 7 days, on average. In addition, the risk for thrombocytopenia after MMR vaccination is several-fold lower than after wild-type rubella (estimated as 1 case per 3000 infections) [39]. Persons with a history of thrombocytopenia or thrombocytopenic purpura might be at increased risk for clinically significant thrombocytopenia after MMR vaccination; thus, a history of these conditions is a precaution for MMR vaccination.

Anaphylaxis and other immediate hypersensitivity reactions can occur after MMR vaccination, and are likely related to allergies to the gelatin or neomycin components of the vaccine [40–42]. In a large population-based study, the risk of anaphylaxis following MMR vaccine was estimated to be 5.14 per million vaccine doses (95% confidence interval, 1.06–15.01) [43]. History of severe allergic reaction to any component of the vaccine is a contraindication to MMR vaccination [28].

Immunocompromised patients should also not receive the MMR vaccine. Potential fatal adverse events in immunocompromised hosts include measles pneumonia, MIBE, and disseminated measles infection [44–47]. This recommendation is inclusive of persons with human immunodeficiency virus (HIV) infection who are severely immunocompromised. However, a systematic review of 28 safety studies of MMR vaccination among HIV-infected children found that adverse events and deaths after measles vaccines were uncommon in this population [48]; thus, persons with HIV infection should receive MMR vaccine if they are not severely immunosuppressed (eg, T-lymphocyte percentage >15% at any age or CD4 cell count >200/µL for those >5 years of age) [28].

MMR vaccine is contraindicated in pregnancy owing to theoretical concerns of fetal harm including congenital rubella syndrome [28]. Nevertheless, a systematic review of vaccines given to pregnant women found that among 4918 pregnant women who inadvertently received MMR vaccine while pregnant, no cases of congenital rubella syndrome were reported [49]. Similarly, a safety review of 131 reports to the Vaccine Adverse Event Reporting System of MMR vaccine administered to pregnant women found that most vaccines were given to women early in pregnancy (when they were unaware of their pregnancy), and in the majority of reports, no adverse events were reported [50]. The highly favorable safety profile of MCV has been an essential component of the global measles eradication strategy.

## MEASLES VACCINE USE AND EFFECTIVENESS

All 194 countries have added MCV into their routine childhood immunization programs, a critical step toward global measles eradication. MCV is usually delivered as a combined multiantigen vaccine, either MR or MMR vaccine. Combination vaccines are cost-effective, in that the addition of other antigens increases cost by much smaller margins compared with the substantial costs incurred by administration, delivery, and wastage of multiple vaccines. Currently, 122 countries have introduced MMR into their routine immunization schedules, with MMR being used solely in most countries in the European and Americas regions [51].

The effectiveness of the measles component of the MMR vaccine is high, 93% after 1 dose and 97% after 2 doses in persons aged ≥12 months. Duration of immunity is likely lifelong after 2 doses [28]. Although the incremental vaccine effectiveness between 1 and 2 doses may seem small, the measles herd immunity threshold is high (>92%), necessitating implementation of a second dose for optimal measles control and elimination to be successfully achieved. As a result of numerous measles outbreaks occurring in vaccinated school-aged children in the United States during the 1980s, a second dose of MMR was recommended for school-aged and college students in 1989. The assimilation of a 2-dose recommendation into the US childhood immunization schedule, in addition to school-entry immunization requirements, the introduction of the Vaccines for Children program in 1993 to improve vaccine access, and concerted efforts in the 1990s by other countries in the Americas region to reduce measles cases and outbreaks (which limited the number of introductions of measles from these countries), ultimately led to the elimination of measles in the United States by 2000.

Timing and delivery strategies for MCV vaccination vary by country and are contingent on multiple factors, including the infrastructure to implement routine immunizations and mass vaccination campaigns, the capacity and preparedness for rapid outbreak response, and the local epidemiology of measles based on surveillance data. Overall disease burden and age at highest risk of disease is often a primary consideration when establishing an immunization schedule. Currently, 41% of countries with measles vaccination programs begin vaccinating infants before 1 year of age (usually at 9 months), owing to the high disease burden in infants.

The chance of exposure to measles for infants and young children in each country must be carefully weighed against the age-specific immunogenicity of each dose of measles vaccine in order to define the optimal age for routine vaccination. Although infants are at risk of severe disease and serious complications, studies have suggested that vaccination at <12 months of age can lead to suboptimal immune responses that continue to be low despite additional doses [52, 53, 54]. In the United States, where exposure to measles is rare, the first dose is recommended at 12–15 months of age and the second dose at 4–6 years, in order to effectively protect young children and to ensure they are fully vaccinated before school entry. Other countries that have achieved measles elimination recommend different schedules (eg, both Canada and Australia recommend the first dose of MMR at 12 months and the second at 18 months of age), highlighting that even in countries with similar measles epidemiology, country-specific healthcare delivery systems play a critical role in establishing the optimal timing for vaccination [39].

## MEASLES VACCINATION COVERAGE

**Figure 2.**
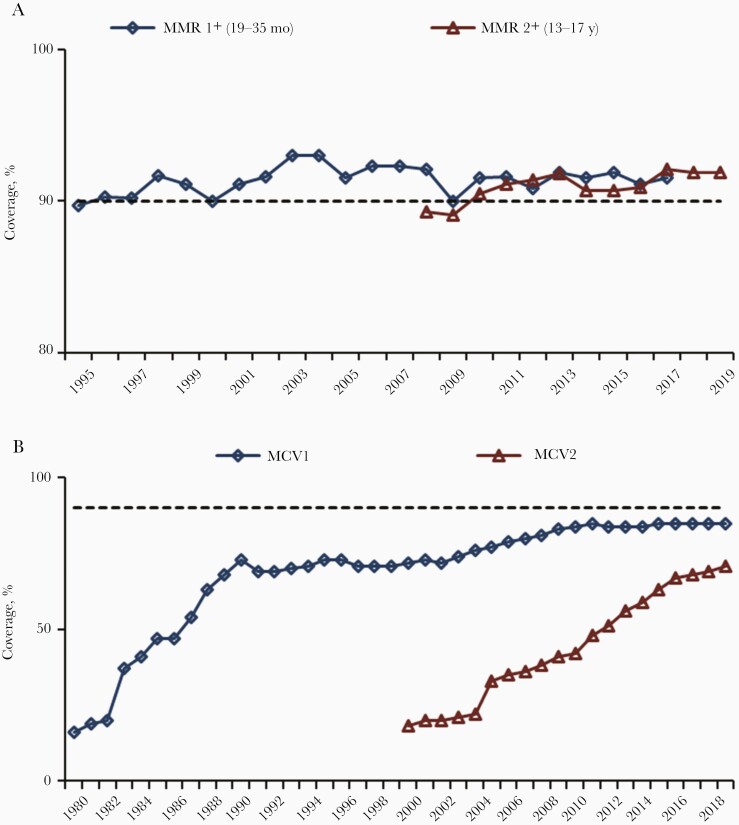
*A,* Estimated measles-mumps-rubella (MMR) vaccination coverage among children aged 19–35 months or 13–17 years. (Data from National Immunization Surveys, United States, 1995–2019; available at https://www.cdc.gov/vaccines/imz-managers/coverage/childvaxview/data-reports/mmr/trend/index.html and https://www.cdc.gov/vaccines/imz-managers/coverage/teenvaxview/data-reports/mmr/trend/index.html). *B,* Estimated measles-containing-vaccine (MCV) first dose (MCV1) and MCV second dose (MCV2) coverage (worldwide data from World Health Organization, 1980–2019; available at http://www.who.int/immunization/monitoring_surveillance/data/en). *A, B,* Horizontal dashed lines represent 90% vaccination coverage.

During 2000–2019, estimated coverage with the routine first-dose MCV (MCV1) increased globally from 72% to 85% (Figure 2), and the number of countries with ≥90% MCV1 coverage increased from 86 (45%) to 122 (63%) [55]. Among countries with ≥90% MCV1 coverage nationally, those that also had ≥80% MCV1 coverage in all districts increased from 1% in 2003 to 22% in 2019. From 2000 to 2019, the number of countries providing a second dose of MCV (MCV2) nationally through routine immunization services increased from 95 (50%) to 177 (91%), and estimated global MCV2 coverage increased from 18% to 71%. However, as of 2019, 17 countries had yet to introduce MCV2 nationally, and MCV1 coverage has remained at 84%–85% globally since 2010. Therefore, mass vaccination campaigns remain a necessary strategy for eliminating measles in many countries. In 2019, approximately 204 million persons received MCV during supplementary immunization activities in 55 countries, and an additional 9 million received MCV during measles outbreak response activities.

Countries that have achieved measles elimination have invested heavily in routine immunization programs to ensure consistently high vaccination coverage against measles and other vaccine-preventable diseases. In the United States, after the first licensure of measles vaccine in 1963 and MMR in 1971, MCV coverage steadily increased, with almost 20 million doses distributed in 1989 alone [20]. National vaccination coverage surveys including MMR were implemented beginning in 1994 for young children and 2006 for adolescents; estimates have remained steady at ≥90% for both 1 and 2 doses since 2010 (Figure 2). In 2017, MMR vaccination coverage was reported to be 91.5% for children aged 19–35 months vaccinated with ≥1 dose and 94.3% for children in kindergarten with ≥2 doses [56, 57]. Similarly, in Canada and Australia, the estimated MCV1 coverage among 2-year-olds in 2017 was 90% and 90.5%, respectively [58, 59].

## ECONOMIC BENEFITS OF MEASLES VACCINATION

The economic benefits of investing in vaccines, particularly measles vaccines, are well established [60–64]. Even in countries with low disease incidence, periodic measles outbreaks continue to occur, causing costly disruptions to society and requiring resource-intensive outbreak response activities [65]. A review of cost estimates of 11 measles outbreaks during the postelimination era in the United States, found that measles costs public health and healthcare institutions a median of approximately $33 000 (US dollars) per case, and $4000 per day of investigation [66]. Furthermore, after accounting for broad economic benefits, the overall societal return on investment for 10 vaccines is 44 (range, 27–67) times the cost of the vaccines, supply chains, and vaccine delivery [60]; the measles vaccine has the highest return on investment, 58 (28–105) times the cost [60]. In the United States, it has been estimated that routine measles vaccination of the 2009 birth cohort prevented 3.8 million measles illnesses and >3000 measles-related deaths, a net savings of more than >$3 billion in direct costs and $8 billion in societal costs [67]. Achieving an eventual measles eradication goal would have massive economic implications and could save current ongoing annual costs of >$2 billion in measles treatment and >15 million disability-adjusted life-years, valued at >$63 billion globally each year [68].

## BARRIERS TO ACHIEVING AND MAINTAINING MEASLES ELIMINATION AND FUTURE CONSIDERATIONS

Despite significant progress in decreasing measles incidence and mortality rates globally since 2000, measles elimination efforts have encountered setbacks in recent years. Estimated MCV1 coverage worldwide has plateaued at 84%–85% for nearly a decade and reported global measles cases increased from a historic low of 132 490 in 2016 to 869 770 in 2019 (556% increase) [55]. Among persons with confirmed measles cases reported to WHO during 2013–2018, 45% were reported to have never received MCV, and 30% had an unknown vaccination history [69].

Several countries lost their elimination status in 2019. In the Americas, the first region of the WHO to have been declared free of measles in 2016, recent reestablishment of endemic virus transmission in Venezuela and Brazil led to a loss of regional elimination. The United States has similarly experienced several sizeable outbreaks following measles importations, mostly in settings with low vaccination coverage, including communities in Ohio [70], Minnesota [71], and Washington [72]. During 2018 and 2019, prolonged outbreaks of almost 1-year duration in undervaccinated communities in New York threatened the measles elimination status of the United States. The 1282 measles cases reported in the United States in 2019 was the highest annual number of reported cases since 1992 [73, 74]. Since 2001, 88% of US residents with confirmed measles cases are either unvaccinated or have an unknown vaccination status [12].

The Measles & Rubella Initiative (M&RI), a global partnership formed in 2001, coordinates efforts to achieve a world without measles and rubella. The M&RI is led by the American Red Cross, the United Nations Foundation, WHO, United Nations Children’s Fund (UNICEF), and the US Centers for Disease Control and Prevention. Since 2001, it has invested >$1.2 billion for measles and rubella elimination efforts. In 2012, a Measles and Rubella Strategic Plan was released covering the period 2012–2020 and was endorsed by the M&RI [75]. Among its goals were reaching measles elimination in ≥5 of the 6 WHO regions, establishing a target date for measles eradication, and achieving a ≥95% coverage with MCV in all districts of all countries no later than 2020. While considerable progress in measles control has been made, none of those targets have been met.

There are multiple challenges to achieving and maintaining the measles herd immunity threshold needed for measles eradication, estimated generally to be >92%–94%. Achieving such immunity levels requires substantial political will, elimination of financial and physical access barriers to measles vaccination, strengthening of public health infrastructure (eg, inconvenient clinic locations and hours), and combating dissemination of misinformation eroding trust and confidence in vaccines [11, 76, 77]. A midterm review of the Measles and Rubella Strategic Plan concluded that measles could be eradicated and offered a number of recommendations to try to support eradication [78]. Notably, vaccine hesitancy was listed by WHO in 2019 as among the top 10 challenges to global health [79]. Both the Centers for Disease Control and Prevention and WHO have established comprehensive initiatives to address vaccine hesitancy [80, 81].

Intensified efforts and resource commitments by global partners and countries are needed to get back on track toward measles elimination. A new immunizations guidance document, the Immunization Agenda 2030 (IA2030) [82], cocreated by WHO and partners and to be endorsed by the World Health Assembly, builds on lessons learned and progress made toward the Global Vaccine Action Plan goals. The IA2030 aims to use measles, a proven effective surrogate or marker for the performance of Expanded Programmes on Immunization [83], to drive efforts to strengthen immunizations and primary healthcare systems [82].

Focusing on measles elimination strategies can enhance delivery of routine immunization for other vaccine-preventable diseases, help identify unvaccinated or undervaccinated communities and close immunity gaps, lead to improvements in surveillance and expansion of cold-chain capacity, create opportunities to provide refresher training on vaccination to healthcare workers, and advance the adoption of strategies used for measles elimination to ensure high coverage for vaccines against other diseases (eg, school-entry requirements) [84]. The IA2030 thus provides an opportunity to strengthen vaccination programs, build on public health partnerships, and leverage data-driven approaches that use disease surveillance to increase vaccination coverage and equity in all communities [65, 85].

## KEY INNOVATIONS FOR MEASLES CONTROL IN RESOURCE-LIMITED COUNTRIES

Innovative approaches for measles diagnostics and vaccination methods, principally to address challenges in resource-limited settings lacking strong healthcare infrastructures, could facilitate the control of measles and help overcome critical barriers to achieving measles elimination.

### New Rapid Diagnostic Test

Because many of the typical clinical signs of measles can also be caused by other infectious agents, including rubella virus, laboratory confirmation of measles is a critical component of the measles control strategy but may not always be feasible in resource-limited settings. Detection of measles-specific IgM antibodies by enzyme immunoassay is the most common method used for case confirmation, though detection of viral RNA by reverse-transcription polymerase chain reaction is increasing in many countries. While most of the IgM tests are performed with serum samples, the use of alternative samples, such as dried blood spots and oral fluid samples, has helped to expand laboratory surveillance. The recent development of a rapid diagnostic test that can provide results in <20 minutes to detect measles IgM in field settings, will facilitate rapid detection of cases and response activities in resource-limited settings where the logistics of sample transport and storage are often challenging [86, 87].

### Alternative Vaccine Delivery Approaches

Alternative vaccine delivery methods that eliminate the need for cold-chain transportation and hypodermic needle and syringe subcutaneous injection could improve vaccine delivery in resource-limited settings and increase vaccination coverage and equity. Several routes of administration have been evaluated. A measles vaccine delivered by the respiratory route as an aerosol was immunogenic but the seroconversion rate was inferior to the rate observed after subcutaneous injection [88]. A dry powder was immunogenic in nonhuman primates, but work was discontinued after a phase I clinical trial [89, 90]. Measles vaccination via the intradermal route produced lower seroresponses because efficient and reliable delivery methods were not available [91]. However, the recent success in development and testing of dissolving microneedle patches for the delivery of measles and rubella vaccine shows that intradermal vaccination by this method is a promising way to improve the efficiency of delivery of vaccination [92, 93].

## CONCLUSIONS

Although measles is one of only a handful of human pathogens that could be eradicated, and despite the broad availability of an intervention tool that could make this possible—an effective and inexpensive measles vaccine with decades of use and an excellent safety track record—the disease remains a leading cause of childhood disease and death globally. Several tough challenges to increasing measles vaccination coverage remain, including poor access to immunization services and dissemination of misinformation eroding vaccine confidence.

Exacerbating these challenges are the recent diversions of public health resources to combat the novel coronavirus disease 2019 pandemic, which have affected routine immunization services and vaccine-preventable disease surveillance in many countries, thereby increasing the risk for outbreaks of measles during or after the pandemic. Yet, the notable successes of measles vaccination in the control and elimination of measles in various regions across the globe undoubtedly demonstrate that these barriers can be overcome, and that measles eradication is attainable. With renewed efforts and resource commitments to achieve elimination, and the implementation of strategies that improve primary health delivery systems, decrease missed opportunities for vaccination and counteract vaccine hesitancy, and incorporate innovative approaches for measles diagnostics and vaccine delivery, a world without measles could be within our grasp.

## Supplementary Data

Supplementary materials are available at *The Journal of Infectious Diseases* online. Consisting of data provided by the authors to benefit the reader, the posted materials are not copyedited and are the sole responsibility of the authors, so questions or comments should be addressed to the corresponding author.

The complete references are available as online Supplemental Material.

## Supplementary Material

jiaa793_suppl_Supplementary-Material

## Notes

***Supplement sponsorship.*** This supplement is sponsored by the Bill and Melinda Gates Foundation.

***Potential conflicts of interest.*** All authors: No reported conflicts. All authors have submitted the ICMJE Form for Disclosure of Potential Conflicts of Interest. Conflicts that the editors consider relevant to the content of the manuscript have been disclosed.
