# Supplementary material for: Measles in the 21st Century: Progress Toward Achieving and Sustaining Elimination
Source: J Infect Dis. 2021 Sep 30;224(Suppl 4):S420–8. doi: 10.1093/infdis/jiaa793 (PMC8482021; doi:10.1093/infdis/jiaa793)
Supplement: jiaa793_suppl_Supplementary-Material [file jiaa793_suppl_supplementary-material.docx]

1. Clements CL, Hussey GD. Measles. In: Murray JL, Lopez AD, Mathers CD, eds. Global epidemiology of infectious diseases. Geneva, Switzerland: World Health Organization, 2004.

2. Hinman AR, Orenstein WA, Bloch AB, et al. Impact of measles in the United States. Rev Infect Dis 1983; 5:439–44.

3. Rota PA, Rota JS, Goodson JL. Subacute sclerosing panencephalitis. Clin Infect Dis 2017; 65:233–4.

4. Laksono BM, de Vries RD, Duprex WP, de Swart RL. Measles pathogenesis, immune suppression and animal models. Curr Opin Virol 2020; 41:31–7.

5. Laksono BM, de Vries RD, Verburgh RJ, et al. Studies into the mechanism of measles-associated immune suppression during a measles outbreak in the Netherlands. Nat Commun 2018; 9:4944.

6. de Swart RL, Ludlow M, de Witte L, et al. Predominant infection of CD150+ lymphocytes and dendritic cells during measles virus infection of macaques. PLoS Pathog 2007; 3:e178.

7. Mina MJ, Kula T, Leng Y, et al. Measles virus infection diminishes preexisting antibodies that offer protection from other pathogens. Science 2019; 366:599–606.

8. Petrova VN, Sawatsky B, Han AX, et al. Incomplete genetic reconstitution of B cell pools contributes to prolonged immunosuppression after measles. Sci Immunol 2019; 4:eaay6125.

9. Wolfson LJ, Grais RF, Luquero FJ, Birmingham ME, Strebel PM. Estimates of measles case fatality ratios: a comprehensive review of community-based studies. Int J Epidemiol 2009; 38:192–205.

10. Salama P, Assefa F, Talley L, Spiegel P, van Der Veen A, Gotway CA. Malnutrition, measles, mortality, and the humanitarian response during a famine in Ethiopia. JAMA 2001; 286:563–71.

11. Patel M, Dumolard L, Nedelec Y, et al. Progress toward regional measles elimination–worldwide, 2000–2018. MMWR Morb Mortal Wkly Rep 2019; 68:1105–11.

12. Clemmons NS, Wallace GS, Patel M, Gastanaduy PA. Incidence of measles in the United States, 2001–2015. JAMA 2017; 318:1279–81

13. Lee AD, Clemmons NS, Patel M, Gastanaduy PA. International importations of measles virus into the United States during the post-elimination era, 20012016. J Infect Dis 2019; 219:1616–23.

14. Roush SW, Murphy TV; Vaccine-Preventable Disease Table Working Group. Historical comparisons of morbidity and mortality for vaccine-preventable diseases in the United States. JAMA 2007; 298:2155–63.

15. World Health Organization. Meeting of the strategic advisory group of experts on immunization, November 2010—summary, conclusions and recommendations. Wkly Epidemiol Rec 2011; 86:1–16.

16. Rota PA, Moss WJ, Takeda M, de Swart RL, Thompson KM, Goodson JL. Measles. Nat Rev Dis Primers 2016; 2:16049.

17. Taylor MJ, Godfrey E, Baczko K, ter Meulen V, Wild TF, Rima BK. Identification of several different lineages of measles virus. J Gen Virol 1991; 72:83–8.

18. Rota PA, Brown K, Mankertz A, et al. Global distribution of measles genotypes and measles molecular epidemiology. J Infect Dis 2011; 204:S514–23.

19. Enders JF, Peebles TC. Propagation in tissue cultures of cytopathogenic agents from patients with measles. Proc Soc Exp Biol Med 1954; 86:277–86.

20. Strebel PM, Papania MJ, Gastanaduy PA, Goodson JL. Measles vaccines. In: Plotkin SA, Orenstein WA, Offit PA, eds. Vaccines. 7th ed. New York, NY: Saunders Elsevier, 2018:579–618.

21. Bankamp B, Takeda M, Zhang Y, Xu W, Rota PA. Genetic characterization of measles vaccine strains. J Infect Dis 2011; 204:S533–48.

22. Griffin DE. Measles vaccine. Viral Immunol 2018; 31:86–95.

23. Greenwood KP, Hafiz R, Ware RS, Lambert SB. A systematic review of human-to-human transmission of measles vaccine virus. Vaccine 2016; 34:2531–6.

24. Tamin A, Rota PA, Wang ZD, Heath JL, Anderson LJ, Bellini WJ. Antigenic analysis of current wild type and vaccine strains of measles virus. J Infect Dis 1994; 170:795–801.

25. Beaty SM, Lee B. Constraints on the genetic and antigenic variability of measles virus. Viruses 2016; 8:109.

26. Chen RT, Markowitz LE, Albrecht P, et al. Measles antibody: reevaluation of protective titers. J Infect Dis 1990; 162:1036–42.

27. Albrecht P, Herrmann K, Burns GR. Role of virus strain in conventional and enhanced measles plaque neutralization test. J Virol Methods 1981; 3:251–60.

28. McLean HQ, Fiebelkorn AP, Temte JL, Wallace GS; Centers for Disease Control and Prevention. Prevention of measles, rubella, congenital rubella syndrome, and mumps, 2013: summary recommendations of the Advisory Committee on Immunization Practices (ACIP). MMWR Recomm Rep 2013; 62:1–34.

29. Peltola H, Heinonen OP. Frequency of true adverse reactions to measles-mumps-rubella vaccine. A double-blind placebo-controlled trial in twins. Lancet 1986; 1:939–42.

30. Weibel RE, Carlson AJ Jr, Villarejos VM, Buynak EB, McLean AA, Hilleman MR. Clinical and laboratory studies of combined live measles, mumps, and rubella vaccines using the RA 27/3 rubella virus. Proc Soc Exp Biol Med 1980; 165:323–6.

31. Rowlands DF, Freestone DS. Vaccination against rubella of susceptible schoolgirls in Reading. J Hyg (Lond) 1971; 69:579–86.

32. Dos Santos BA, Ranieri TS, Bercini M, et al. An evaluation of the adverse reaction potential of three measles-mumps-rubella combination vaccines. Rev Panam Salud Publica 2002; 12:240–6.

33. LeBaron CW, Bi D, Sullivan BJ, Beck C, Gargiullo P. Evaluation of potentially common adverse events associated with the first and second doses of measles-mumps-rubella vaccine. Pediatrics 2006; 118:1422–30.

34. Committee to Review Adverse Effects of Vaccines, Institute of Medicine; Stratton K, Ford A, Rusch E, Clayton EW, eds. Adverse effects of vaccines: evidence and causality. Washington, DC: National Academies Press, 2011.

35. Barlow WE, Davis RL, Glasser JW, et al; Centers for Disease Control and Prevention Vaccine Safety Datalink Working Group. The risk of seizures after receipt of whole-cell pertussis or measles, mumps, and rubella vaccine. N Engl J Med 2001; 345:656–61.

36. Klein NP, Fireman B, Yih WK, et al; Vaccine Safety Datalink. Measles-mumps-rubella-varicella combination vaccine and the risk of febrile seizures. Pediatrics 2010; 126:e1–8.

37. Klein NP, Lewis E, Baxter R, et al. Measles-containing vaccines and febrile seizures in children age 4 to 6 years. Pediatrics 2012; 129:809–14.

38. France EK, Glanz J, Xu S, et al; Vaccine Safety Datalink Team. Risk of immune thrombocytopenic purpura after measles-mumps-rubella immunization in children. Pediatrics 2008; 121:e687–92.

39. Bayer WL, Sherman FE, Michaels RH, Szeto IL, Lewis JH. Purpura in congenital and acquired rubella. N Engl J Med 1965; 273:1362–6.

40. Pool V, Braun MM, Kelso JM, et al; VAERS Team. US Vaccine Adverse Event Reporting System. Prevalence of anti-gelatin IgE antibodies in people with anaphylaxis after measles-mumps rubella vaccine in the United States. Pediatrics 2002; 110:e71.

41. James JM, Burks AW, Roberson PK, Sampson HA. Safe administration of the measles vaccine to children allergic to eggs. N Engl J Med 1995; 332:1262–6.

42. Rietschel RL, Bernier R. Neomycin sensitivity and the MMR vaccine. JAMA 1981; 245:571.

43. McNeil MM, Weintraub ES, Duffy J, et al. Risk of anaphylaxis after vaccination in children and adults. J Allergy Clin Immunol 2016; 137:868–78.

44. Monafo WJ, Haslam DB, Roberts RL, Zaki SR, Bellini WJ, Coffin CM. Disseminated measles infection after vaccination in a child with a congenital immunodeficiency. J Pediatr 1994; 124:273–6.

45. Mawhinney H, Allen IV, Beare JM, et al. Dysgammaglobulinaemia complicated by disseminated measles. Br Med J 1971; 2:380–1.

46. Centers for Disease Control and Prevention. Measles pneumonitis following measles-mumps-rubella vaccination of a patient with HIV infection, 1993. MMWR Morb Mortal Wkly Rep 1996; 45:603–6.

47. Angel JB, Walpita P, Lerch RA, et al. Vaccine-associated measles pneumonitis in an adult with AIDS. Ann Intern Med 1998; 129:104–6.

48. Scott P, Moss WJ, Gilani Z, Low N. Measles vaccination in HIV-infected children: systematic review and metaanalysis of safety and immunogenicity. J Infect Dis 2011; 204:S164–78.

49. Nasser R, Rakedzon S, Dickstein Y, et al. Are all vaccines safe for the pregnant traveller? a systematic review and meta-analysis. J Travel Med 2020; 27:taz074.

50. Sukumaran L, McNeil MM, Moro PL, Lewis PW, Winiecki SK, Shimabukuro TT. Adverse events following measles, mumps, and rubella vaccine in adults reported to the Vaccine Adverse Event Reporting System (VAERS), 2003–2013. Clin Infect Dis 2015; 60:e58–65.

51. World Health Organization. WHO vaccine-preventable diseases: monitoring system. 2018 global summary http://apps.who.int/immunization_monitoring/globalsummary/schedules. Accessed 1 June 2020.

52. Brinkman ID, de Wit J, Smits GP, et al. Early measles vaccination during an outbreak in the Netherlands: short-term and long-term decreases in antibody responses among children vaccinated before 12 months of age. J Infect Dis 2019; 220:594–602.

53. Gans HA, Arvin AM, Galinus J, Logan L, DeHovitz R, Maldonado Y. Deficiency of the humoral immune response to measles vaccine in infants immunized at age 6 months. JAMA 1998; 280:527–32.

54. Gans HA, Yasukawa LL, Alderson A, et al. Humoral and cell-mediated immune responses to an early 2-dose measles vaccination regimen in the United States. J Infect Dis 2004; 190:83–90.

55. Patel MK, Goodson JL, Alexander JP, Jr., et al. Progress toward regional measles elimination–worldwide, 2000–2019. MMWR Morb Mortal Wkly Rep 2020; 69:1700–5.

56. Hill HA, Elam-Evans LD, Yankey D, Singleton JA, Kang Y. Vaccination coverage among children aged 19–35 months—United States, 2017. MMWR Morb Mortal Wkly Rep 2018; 67:1123–8.

57. Mellerson JL, Maxwell CB, Knighton CL, Kriss JL, Seither R, Black CL. Vaccination coverage for selected vaccines and exemption rates among children in kindergarten—United States, 2017-18 school year. MMWR Morb Mortal Wkly Rep 2018; 67:1115–22.

58. Government of Canada. Highlights from the 2017 childhood National Immunization Coverage Survey (cNICS). https://www.canada.ca/en/services/health/publications/vaccines-immunization/vaccine-uptake-canadian-childrenpreliminary-results-2017-childhood-national-immunizationcoverage-survey.html. Accessed 13 September 2020.

59. Australian Government Department of Health. Historical coverage data tables for all children. https://www.health.gov.au/health-topics/immunisation/childhoodimmunisation-coverage/historical-coverage-data-tablesfor-all-children#2-year-olds. Accessed 13 September 2020.

60. Ozawa S, Clark S, Portnoy A, Grewal S, Brenzel L, Walker D. Return on investment from childhood immunization in low- and middle-income countries, 2011–20. Health Aff 2016; 35:199–207.

61. Lee LA, Franzel L, Atwell J, et al. The estimated mortality impact of vaccinations forecast to be administered during 2011–2020 in 73 countries supported by the GAVI Alliance. Vaccine 2013; 31:B61– 72.

62. Ozawa S, Clark S, Portnoy A, et al. Estimated economic impact of vaccinations in 73 low- and middle-income countries, 2001–2020. Bull World Health Organization 2017; 95:629–38.

63. Durrheim DN. Measles eradication—retreating is not an option. Lancet Infect Dis 2020; 20:e138-e141

64. Ozawa S, Yemeke TT, Thompson KM. Systematic review of the incremental costs of interventions that increase immunization coverage. Vaccine 2018; 36:3641–9.

65. Durrheim DN, Crowcroft NS. The price of delaying measles eradication. Lancet Public Health 2017; 2:e130–1.

66. Pike J, Leidner AJ, Gastanaduy PA. A review of measles outbreak cost estimates from the US in the post-elimination era (2004–2017): estimates by perspective and cost type. Clin Infect Dis 2020; 71:1568–76.

67. Zhou F, Shefer A, Wenger J, et al. Economic evaluation of the routine childhood immunization program in the United States, 2009. Pediatrics 2014; 133:577–85.

68. Thompson KM, Odahowski CL. Systematic review of health economic analyses of measles and rubella immunization interventions. Risk Anal 2016; 36:1297–314.

69. Patel MK, Antoni S, Nedelec Y, et al. The changing global epidemiology of measles, 2013–2018. J Infect Dis 2020; 222:1117–28.

70. Gastañaduy PA, Budd J, Fisher N, et al. A measles outbreak in an underimmunized Amish community in Ohio. N Engl J Med 2016; 375:1343–54.

71. Banerjee E, Griffith J, Kenyon C, et al. Containing a measles outbreak in Minnesota, 2017: methods and challenges. Perspect Public Health 2020; 140:162–71.

72. Carlson A, Riethman M, Gastañaduy P, et al. Notes from the field: community outbreak of measles—Clark County, Washington, 2018-2019. MMWR Morb Mortal Wkly Rep 2019; 68:446–7.

73. Patel M, Lee AD, Redd SB, et al. Increase in measles cases–United States, January 1-April 26, 2019. MMWR Morb Mortal Wkly Rep 2019; 68:402–4.

74. Patel M, Lee AD, Clemmons NS, et al. National update on measles cases and outbreaks—United States, January 1-October 1, 2019. MMWR Morb Mortal Wkly Rep 2019; 68:893–6.

75. World Health Organization. 2012–2020 Global measles and rubella strategic plan. https://apps.who.int/iris/bitstream/handle/10665/44855/9789241503396_eng.pdf;jsessionid=E46691BCFE3715F748D48129CCD14A30?sequence=1.Accessed 10 June 2020.

76. Gostin LO, Hodge JG Jr, Bloom BR, et al. The public health crisis of underimmunisation: a global plan of action. Lancet Infect Dis 2020; 20:e11–6.

77. Peck M, Gacic-Dobo M, Diallo MS, Nedelec Y, Sodha SV, Wallace AS. Global routine vaccination coverage, 2018. Morb Mortal Wkly Rep 2019; 68:937–42.

78. Orenstein WA, Hinman A, Nkowane B, Olive JM, Reingold A. Measles and Rubella Global Strategic Plan 2012–2020 midterm review. Vaccine 2018; 36:A1–34.

79. World Health Organization. Ten threats to global health in 2019. https://www.who.int/vietnam/news/feature-stories/detail/ten-threats-to-global-health-in-2019. Accessed 5 September 2020.

80. Centers for Disease Control and Prevention. Vaccinate with confidence. https://www.cdc.gov/vaccines/partners/vaccinate-with-confidence.html. Accessed 8 September 2020.

81. World Health Organization. Improving vaccination demand and addressing hesitancy. https://www.who.int/immunization/programmes_systems/vaccine_hesitancy/en/. Accessed 8 September 2020.

82. World Health Organization. Immunization Agenda 2030: a global strategy to leave no one behind. Geneva, Switzerland: World Health Organization. 2018. https://www.who.int/teams/immunization-vaccines-and-biologicals/strategies/ia2030. Accessed 6 May 2020.

83. Goodson JL, Alexander JP, Linkins RW, Orenstein WA. Measles and rubella elimination: learning from polio eradication and moving forward with a diagonal approach. Expert Rev Vaccines 2017; 16:1203–16.

84. Biellik RJ, Orenstein WA. Strengthening routine immunization through measles-rubella elimination. Vaccine 2018; 36:5645–50.

85. Durrheim DN. Measuring equitable delivery of vaccines. Vaccine 2020; 38:2433–4.

86. Warrener L, Slibinskas R, Chua KB, et al. A point-of-care test for measles diagnosis: detection of measles-specific IgM antibodies and viral nucleic acid. Bull World Health Organ 2011; 89:675–82.

87. Shonhai A, Warrener L, Mangwanya D, et al. Investigation of a measles outbreak in Zimbabwe, 2010: potential of a point of care test to replace laboratory confirmation of suspected cases. Epidemiol Infect 2015; 143:3442–50.

88. Low N, Bavdekar A, Jeyaseelan L, et al. A randomized, controlled trial of an aerosolized vaccine against measles. N Engl J Med 2015; 372:1519–29.

89. Cape S, Chaudhari A, Vaidya V, et al; MA Group. Safety and immunogenicity of dry powder measles vaccine administered by inhalation: a randomized controlled Phase I clinical trial. Vaccine 2014; 32:6791–7.

90. Klinkenberg D, Nishiura H. The correlation between infectivity and incubation period of measles, estimated from households with two cases. J Theor Biol 2011; 284:52–60.

91. Cutts FT, Clements CJ, Bennett JV. Alternative routes of measles immunization: a review. Biologicals 1997; 25:323–38.

92. Edens C, Collins ML, Ayers J, Rota PA, Prausnitz MR. Measles vaccination using a microneedle patch. Vaccine 2013; 31:3403–9.

93. Joyce JC, Carroll TD, Collins ML, et al. A microneedle patch for measles and rubella vaccination is immunogenic and protective in infant rhesus macaques. J Infect Dis 2018; 218:124–32.
